# Temporal Data-Driven Sleep Scheduling and Spatial Data-Driven Anomaly Detection for Clustered Wireless Sensor Networks

**DOI:** 10.3390/s16101601

**Published:** 2016-09-28

**Authors:** Gang Li, Bin He, Hongwei Huang, Limin Tang

**Affiliations:** 1 School of Electronics and Information Engineering, Tongji University, Shanghai 201804, China; 1310482@tongji.edu.cn (G.L.); 1333700@tongji.edu.cn (L.T.); 2 Department of Geotechnical Engineering, Tongji University, Shanghai 200092, China; huanghw@tongji.edu.cn

**Keywords:** spatial–temporal correlation, data-driven sleep scheduling, data-driven anomaly detection, WSN

## Abstract

The spatial–temporal correlation is an important feature of sensor data in wireless sensor networks (WSNs). Most of the existing works based on the spatial–temporal correlation can be divided into two parts: redundancy reduction and anomaly detection. These two parts are pursued separately in existing works. In this work, the combination of temporal data-driven sleep scheduling (TDSS) and spatial data-driven anomaly detection is proposed, where TDSS can reduce data redundancy. The TDSS model is inspired by transmission control protocol (TCP) congestion control. Based on long and linear cluster structure in the tunnel monitoring system, cooperative TDSS and spatial data-driven anomaly detection are then proposed. To realize synchronous acquisition in the same ring for analyzing the situation of every ring, TDSS is implemented in a cooperative way in the cluster. To keep the precision of sensor data, spatial data-driven anomaly detection based on the spatial correlation and Kriging method is realized to generate an anomaly indicator. The experiment results show that cooperative TDSS can realize non-uniform sensing effectively to reduce the energy consumption. In addition, spatial data-driven anomaly detection is quite significant for maintaining and improving the precision of sensor data.

## 1. Introduction

Monitoring systems based on wireless sensor networks (WSNs) play an important role in sensor network applications, such as civil structure monitoring [1], environment monitoring, habitat monitoring and military warfare. A large number of sensor nodes are deployed to monitor the object, and their sensor data are transferred to the sink node through single-hop or multi-hop communication in WSNs [2]. Since these sensor nodes are in similar environments, sensor data from nodes nearby have a high spatial correlation. In addition, a single sensor node has a high temporal correlation when physical phenomena of the object change slowly. Accordingly, sensor data of such monitoring systems have a high spatial–temporal correlation.

Tunnel structural health monitoring is vital to the operation safety of subway systems. The tunnel monitoring system based on WSNd is gradually replacing manual monitoring because of the harsh environment in the tunnel. Compared with monitoring systems based on the cable, the tunnel monitoring system based on WSNs can reduce costs and the complexity of sensor deployment. A fundamental challenge of monitoring systems based on WSNs lies in the energy constraint of battery-powered sensor nodes, which limits the network lifetime. Moreover, this work is based on the monitoring system of the metro tunnel, in which it is difficult to enter. The labor cost of replacing the battery is quite high. Low energy consumption is therefore extremely important in the tunnel monitoring system. A large number of scholars have confirmed that exploiting the spatial–temporal correlation of sensor data is helpful for reducing energy consumption. The energy consumption of sensor nodes is mainly caused by wireless transmission, data sensing and node operation. To reduce the energy consumption caused by wireless transmission, data aggregation based on the spatial–temporal correlation has been proposed [3]. This kind of data aggregation uses the spatial–temporal correlation to reduce the amount of transmitted data. Aimed at reducing the energy consumption caused by data sensing, sparse sensing based on the spatial–temporal correlation can reduce the amount of collected samples [4]. To reduce the energy consumption caused by node operation, the spatial–temporal correlation can be used to realize the sleep scheduling of nodes [5]. In addition, the sleep scheduling can also reduce the amount of collected samples and transmitted data, so that it is a significant method for reducing the energy consumption of sensor nodes. Accordingly, this work focuses on the data-driven sleep scheduling to save the energy.

In this work, the tunnel monitoring system is used in the shield tunnel, which generally takes the form of a ring to distinguish segments. Sensor nodes are therefore arranged in the forms of rings. Besides the aforementioned fundamental challenge about the energy constraint, the tunnel monitoring system has two core requirements for data: (1) synchronous acquisition in the same ring should be realized for analyzing the situation of the every ring; and (2) high precision of all sensor data should be ensured for analyzing and monitoring the situation of the tunnel structure. In consideration of the second core requirement for data, the spatial correlation is used in a proposed anomaly detection, which is termed spatial data-driven anomaly detection. This anomaly detection method can determine whether the node is working properly, which is a necessary precondition for keeping the high precision of all sensor data. On the other hand, temporal correlation is used in the proposed sleep scheduling, which is termed temporal data-driven sleep scheduling. This sleep scheduling works in different nodes in a cooperative way according to the first core requirement for data.

For the tunnel monitoring system, a network topology with a long and linear cluster structure is also built [6]. Clustering has been widely used in WSNs, which can reduce the energy consumption [7]. In clustered WSNs, some sensor nodes become cluster heads to collect data from their respective cluster members, and then forward these data to the sink node. Based on the above, this work presents temporal data-driven sleep scheduling and spatial data-driven anomaly detection for clustered WSNs. The main contributions and novelties of this work are as follows: (1) the combination of temporal data-driven sleep scheduling and spatial data-driven anomaly detection is proposed. The proposed approach is data-driven, and thus it can be used in any clustered WSN; (2) the proposed cooperative temporal data-driven sleep scheduling (TDSS) not only can realize non-uniform sensing to reduce the energy consumption, but also can realize synchronous acquisition in the same ring for analyzing the situation of the every ring; and (3) the proposed spatial data-driven anomaly detection, which generates an anomaly indicator *ξ* to decide the priority level of node maintenance, can maintain and improve the precision of sensor data effectively.

The rest of this work is organized as follows. Section 2 reviews related work. In Section 3, the temporal data-driven sleep scheduling model is proposed. Section 4 describes a cluster-based cooperation mechanism. Performance of the proposed methods is evaluated in Section 5. Section 6 concludes this work.

## 2. Related Work

In most of the existing works, the spatial–temporal correlation is used to reduce data redundancy. Data redundancy can be reduced by several ways including data aggregation, sparse sensing, sleep scheduling and so on. Data aggregation techniques using soft computing methods for WSN mainly have four types including neural-based data aggregation, fuzzy-based data aggregation, genetic-based data aggregation and swarm-based data aggregation [8]. Most of these data aggregation techniques are based on cluster or tree. Alsheikh et al. [9] presented a data compression algorithm with error bound guarantee, where a three-layer neural network was used to extract the feature of data. Their algorithm exploits the spatial–temporal correlation in the raw data to generate a lower-dimensional representation so that the amount of data transmitted by the cluster head is reduced. Since their algorithm is performed in the cluster head, the amount of data transmitted from cluster members to the cluster head is not changed. To further reduce the amount of such data, Arunraja et al. [10] deployed a dual prediction based reporting between cluster members and their cluster head, which is based on the temporal correlation of sensor data. After a few rounds of data transmission, this dual prediction in the cluster member and the cluster head starts to predict the next data from previous data synchronously. If the prediction is in line with the actual data, the cluster member will not transmit the actual data to the cluster head. In addition, the cluster head reduces the spatial data redundancy after receiving data from its cluster members. Although the energy spent for the transmission is further optimized by reducing the amount of data transmitted from cluster members to the cluster head, the energy consumption of sensing is still ignored. Chen et al. [4] realized a distributed adaptive sparse sensing based on compressive sensing, which reduced the amount of collected samples and recovered the missing data by using the spatial–temporal correlation. By exploiting spatial and temporal (spatiotemporal) correlations among sensor readings simultaneously, Gong et al. [11] proposed a spatiotemporal compressive network coding. This scheme compressed and recovered sensor data in a more energy-efficient manner, which was validated in their simulation.

The aforementioned methods based on the spatial–temporal correlation can reduce the energy consumption by optimizing data transmission and data sensing, which need to keep sensor nodes active. Sleep scheduling is therefore considered to further reduce the energy consumption. Ma et al. [12] proposed an optimal sleep scheduling scheme based on balanced energy consumption, where sensor nodes had three states including active, sleep and pre-sleep. Simulation results showed that their sleep scheduling improved the sleep ratio and extended the network lifetime. They focused on balanced energy consumption, which was realized by considering the residual energy and the distance among nodes. Aimed at keeping high network coverage through a minimum number of sensor devices in active state, Al-Kahtani et al. [5] presented an efficient cluster-based sleep scheduling. In their work, the sensing range of the node is larger than the distance between nodes. Thus, some nodes can remain in sleep mode if monitoring areas nearby are covered. These works are based on the existing clustering routing. Yang et al. [13] proposed sleeping multipath routing that could trade off network lifetime and reliability by dynamically activating an optimal number of paths and putting the rest of the sensors to sleep. An adaptive sensor sleeping solution was also proposed, and it further prolonged the network lifetime by putting sensors to sleep at both the routing and the Media Access Control (MAC) layers. Liu et al. [14] presented a joint routing-and-sleep-scheduling scheme by incorporating the design of routing and sleep scheduling into one optimization framework. An asynchronous sleep scheduling mechanism in different sensor nodes was described. The problem about optimal joint routing and sleep scheduling was solved through an iterative geometric programming algorithm. According to existing research results, sleep scheduling is an efficient way to reduce the energy consumption in WSNs.

Some scholars have exploited the spatial–temporal correlation of sensor data to detect the anomaly. Titouna et al. [15] proposed a two-level outlier detection approach using Bayes classifiers, where the first level was implemented locally inside the sensor nodes and the second level was carried out in the cluster head or gateway. Their approach used these two levels based on the spatial–temporal correlation to ensure a high accuracy and reduce the false alarm. To detect faulty readings with greater accuracy, Nisha et al. [16] realized anomaly detection by using a proactive correlated fuzzy system. Their correlation acts included the fuzzy-temporal act, the fuzzy-attribute act, and the fuzzy-spatial act. Their experimental results proved that their method outperformed the existing work in anomaly detection accuracy and false alarm. Xiang et al. [17] focused on the spatial correlation to realize event detection in the three-dimensional space. The Neyman–Pearson principle and collaborative detectors were utilized to improve the accuracy of detecting the occurrence of the event. According to existing research results, anomaly detection based on the spatial–temporal correlation is feasible and valuable.

## 3. Temporal Data-Driven Sleep Scheduling Model

**Figure 1:**
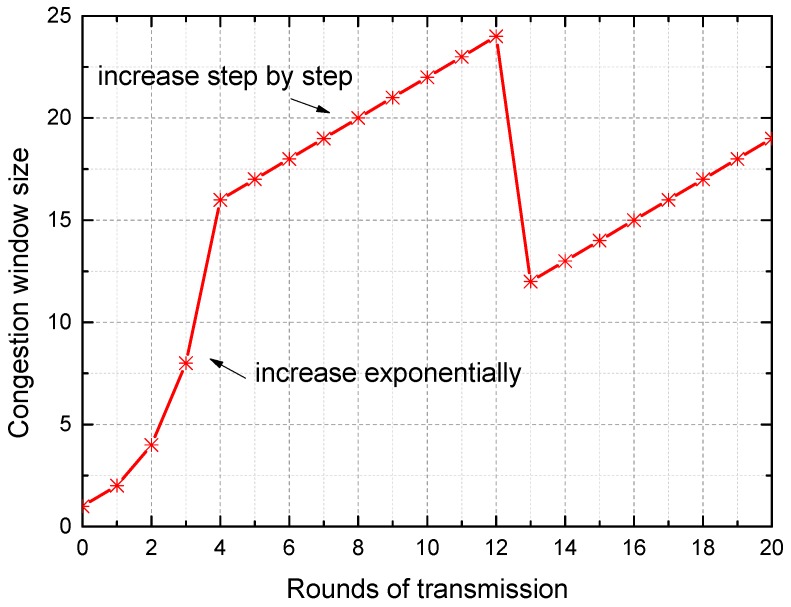
The change in congestion window size of slow starts and the congestion avoidance algorithm.

The TDSS model is inspired by transmission control protocol (TCP) congestion control. For ensuring the stability and efficiency of data transmission in the network, TCP congestion control prevents the occurrence of a situation in which network performance is affected or even paralyzed because some data packets are not processed promptly. The core idea of TCP congestion control is to maximize network throughput without congestion by adjusting the congestion window. The change in congestion window size of slow starts and the congestion avoidance algorithm is shown in Figure 1. In the case of sleep scheduling of sensor nodes, sleep scheduling is just like a kind of “congestion control” of time.

To describe the TDSS model, the following parameters are defined, namely, the maximum sleep time *TS_max_*, the minimum sleep time *TS_min_*, the sleep time step *T*, the counter variable *N* (set to 0 by default), and the threshold *τ*. *TS_max_* and *TS_min_* should be integer multiples of *T*. In this section, the TDSS model is based on a single sensor node. The main operation processes of the TDSS model are as follows: (1) the first sleep time *TS*_1_ is set to *TS_min_*; (2) the absolute value of difference between two sensor data is recorded as ∆*V*. After the first sleep time *TS*_1_, if ∆*V* is less than *τ*, the second sleep time *TS*_2_ will be equal to *TS*_1_ × 2; (3) After the second sleep time *TS*_2_, if ∆*V* is less than *τ*, the third sleep time *TS*_3_ will be equal to *TS*_2_ × 2, and so on; (4) After the *i*th sleep time *TS_i_*, if ∆*V* < *τ* and 2 × *TS_i_* > *TS_max_*, the (*i* + 1)th sleep time *TS_i_*_+1_ will be equal to *TS*_i_ + *T*; (5) After the (*j* − 1)th sleep time *TS_j_*_-1_, if ∆*V* is greater than or equal to *τ*, the *j*th sleep time *TS_j_* will be equal to *TS_j_*_-1_ − *T*, and *N* = *N* + 1; (6) After *TS_j_*, if ∆*V* is still greater than or equal to *τ*, the (*j* + 1)th sleep time *TS_j_*_+1_ will be equal to *TS_j_*/2, and *N* = *N* + 1. Otherwise, if ∆*V* is less than *τ*, *TS_j_*_+1_ will be equal to *TS_j_* + *T*, and *N* = 0; (7) If *N* is greater than 3, the sleep time *TS* will be set to *TS_min_*.
**TDSS Model: Temporal Data-Driven Sleep Scheduling Model**Input: Sensor data of a single sensor node, *TS_max_*, *TS_min_*, *T*, *N* and *τ*.Output: The sleep time *TS*1. Initialize *TS_max_*, *TS_min_*, *T*, *N* = 0 and *τ*.2. Collect the first sensor data, enter the sleeping state with *TS* = *TS_min_*, and then collect the second sensor data.3. while (true)4. while (∆*V* < *τ*)5. *TS* = *TS* × 2, *N* = 06. if *TS > TS_max_*, then7. *TS* = *TS*/2 + *T*8. if *TS > TS_max_*, then9. *TS* = *TS_max_*10. Enter the sleeping state, and then collect sensor data after *TS*11. while (∆*V* ≥ *τ*)12. *N* = *N* + 113. if *N* ≤ 1, then14. *TS* = *TS* − *T*15. else if *N* ≤ 3, then16. *TS* = *TS*/217. else if *N* >3, then18. *TS* = *TS_min_*19. if *TS* < *TS_min_*, then20. *TS* = *TS_min_*21. Enter the sleeping state, and then collect the sensor data after *TS*22. end


**Figure 2:**
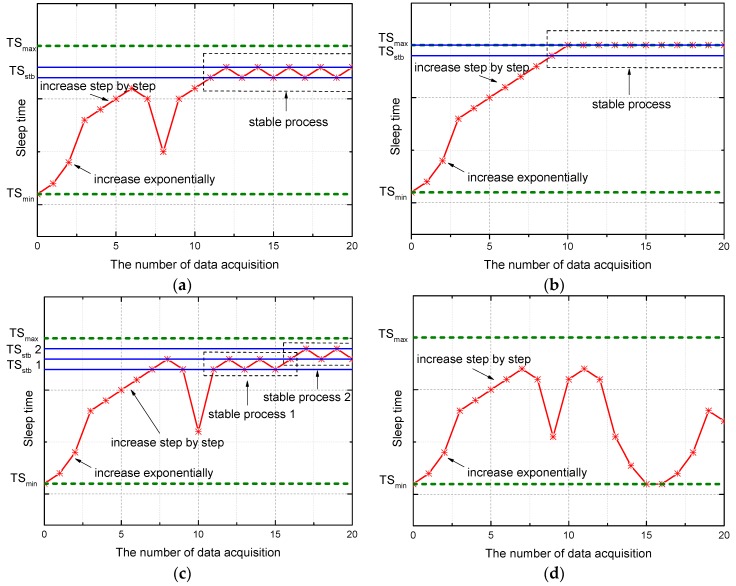
The change in the sleep time of temporal data-driven sleep scheduling (TDSS) with different inputs. (**a**) sinusoidal signal *τ*sin(*ωt*) as the input of TDSS; (**b**) constant value as the input of TDSS; (**c**) inclination of metro tunnel without metro operation; and (**d**) inclination of metro tunnel with metro operation.

For slowly changed data like inclination data, temperature data, splaying amount of joints, and leakage data in the tunnel, the TDSS model can keep the sleep time at a relatively stable value after the sleep time is approaching appropriate sleep time. The stage of achieving the stable sleep time (*TS_stb_*) is termed the stable process in this work. The criterion for entering the stable process is that four consecutive *TS* vary between *X* and *X* + *T* where *X* is a constant. In addition, *X* and *X* + *T* are recorded as *TS_stb_*. This kind of data-driven sleep scheduling model is not confined by the types of sensors. To describe the change in the sleep time of TDSS, four cases are selected as the inputs of TDSS, as shown in Figure 2. When the number of data acquisition is less than 11 in Figure 2a, the stage of approaching the stable sleep time is termed the stability approximation process in this work. TDSS is inspired by TCP congestion control. The stability approximation process is therefore similar to the trend of the change in congestion window size shown in Figure 1. In Figure 2a, a sinusoidal signal *τ*sin(*ωt*) is selected as the input of TDSS. At the beginning of TDSS, ∆*V* is always less than *τ*, and 2 × *TS* ≤ *TS_max_*. Thus, *TS* increases exponentially. If ∆*V* < *τ* and 2 × *TS* > *TS_max_*, TDSS will increase step by step. When ∆*V* is close to *τ*, TDSS enters the stable process. In Figure 2b, where the input is a constant value, ∆*V* is always less than *τ*, and thus *TS* will remain at *TS_max_* after these two stages including “increase exponentially” and “increase step by step”. In Figure 2c, the inclination of metro tunnel without metro operation changes very little, which is caused by the surrounding disturbance and sensor errors. Accordingly, although TDSS enters the stable process temporarily, *TS_stb_* can bee changed according to the variation of sensor data. In Figure 2d, *TS* decreases rapidly after the 11th data acquisition because metro train vibration can cause instantaneous change in the inclination angle. Small *TS* can preserve more data with the key features about instantaneous change in the inclination angle. After the metro train leaves, *TS* increases to reduce the redundancy of sensor data. As stated above, TDSS can enter the stable process when the input of TDSS is constant or changing regularly for a while. The aim of TDSS is reducing the temporal redundancy of sensor data through appropriate sleep time rather than achieving the stable process. To reduce the redundancy of actual sensor data, TDSS achieves appropriate *TS* for actual sensor data instead of the true value, which cannot be obtained. Since actual sensor data have certain randomness caused by random events and errors, TDSS in a practical application does not necessarily tend to stabilize, which is exactly a characteristic of the data-driven mechanism. TDSS can achieve appropriate *TS* according to the variation of actual sensor data to reduce data redundancy, which will be verified in Section 5.

## 4. Cluster-Based Cooperation Mechanism

### 4.1. Long and Linear Cluster Structure

**Figure 3:**
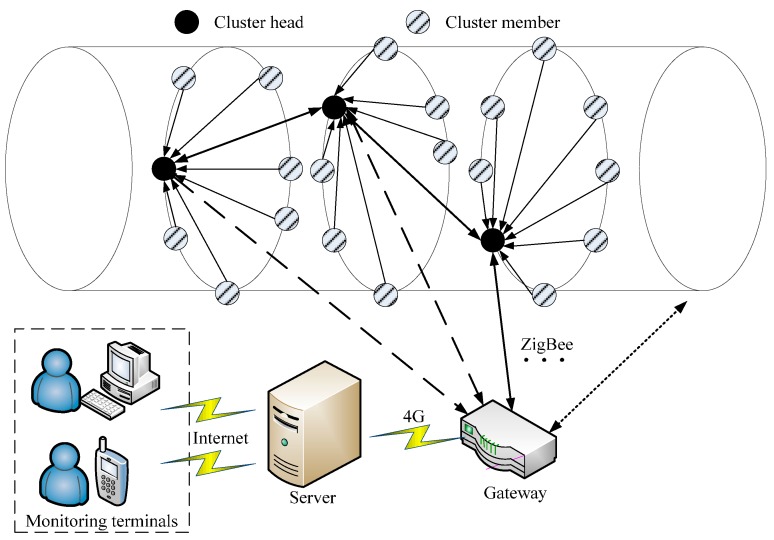
The tunnel monitoring system based on the long and linear cluster structure.

For the tunnel monitoring system, a network topology with long and linear cluster structure is built. Sensor nodes in the same ring form a cluster, and then a long and linear cluster structure is formed in the long and linear tunnel. As shown in Figure 3, a cluster head in every ring collects data from its cluster members. If the gateway is in the communication range of the cluster head, the cluster head will transmit data to the gateway directly; otherwise, the cluster head will transmit data to the gateway through the inter-cluster relay.

The tunnel monitoring system has two core requirements for data, which is described in Section 1. The first core requirement on data is considered in Section 4.2, and the second core requirement for data is considered in Section 4.3. The long and linear cluster structure is the basis of Section 4.2 and Section 4.3. Its brief description is therefore given. The main steps in building long and linear cluster structure are as follows: (1) the round mechanism, which has two stages including the stage of establishing clusters and the stage of stable work, is used in the clustering routing algorithm; (2) the first step of establishing clusters in every round is to select the cluster head. Sensor nodes in the same ring become the cluster head, in turn, so that energy consumption of nodes is balanced; (3) the second step of establishing clusters is to form a cluster. Except for the cluster head, other sensor nodes in the same ring become cluster members of this cluster head. Then, then a cluster is formed; (4) the third step of establishing clusters is to realize multi-hop among clusters by a greedy algorithm [6] and (5) sensor nodes enter the stage of stable work with TDSS.

### 4.2. Cooperative TDSS in the Cluster

The proposed TDSS model in Section 3 is based on a single sensor node. According to the first core requirement for data, cooperative TDSS in the cluster is therefore presented to keep the same sleep time in the same ring.

Since time synchronization error among clusters can be tolerated in the tunnel monitoring system, sensor nodes are configured to be same time before installation, which is a simple way to realize adjustment time among clusters roughly. Sensor data in the same cluster require a higher time synchronization precision. Time synchronization in the cluster is easier to be realized than time synchronization among clusters. Accordingly, reference broadcast synchronization [18], which is commonly used, is used to realize time synchronization in the cluster.

According to the TDSS model, every sensor node has calculated the sleep time *TS*, which is not the final sleep time. Cooperative TDSS in the cluster further processes these sleep time to generate a unified sleep time in the cluster. The *i*th cluster, which has seven sensor nodes *i*_1_, *i*_2_, *i*_3_, *i*_4_, *i*_5_, *i*_6_ and *i*_7_, is analyzed as an example. Firstly, *TS*(*i_x_*) denotes the sleep time of these sensor nodes, which is obtained from the TDSS model. The minimum *TS*(*i_x_*), which is recorded as min(*TS*(*i_x_*)), is selected by the sorting method. Secondly, the unified sleep time in the *i*th cluster, which is recorded as *TS*(*i*), can be calculated as shown in Equation (1):

(1) $$TS\left(i\right)=\text{min}\left(TS\left(i_{x}\right)\right)+\left\lfloor \frac{1}{m}\sum_{j=1}^{m}\frac{TS\left(i_{j}\right)-\text{min}\left(TS\left(i_{x}\right)\right)}{T}\right\rfloor \times T,$$

where *m* denotes the number of sensor nodes in the cluster. In the case of *i*th cluster, *m* is equal to 7.

Every sensor node in the *i*th cluster uses the same sleep time *TS*(*i*). Through the same sleep time and time synchronization in the cluster, synchronous acquisition in the same ring can be realized.

### 4.3. Spatial Data-Driven Anomaly Detection in the Cluster

Besides temporal correlation of sensor data, which is the basis of cooperative TDSS in the cluster, the spatial correlation of sensor data should be considered. Different from most existing works where the spatial correlation is used to further compress data, the spatial correlation is used in cluster-based anomaly detection in this work. Spatial compression can cause some loss of precision, which is against the second core requirement for data, and spatial data-driven anomaly detection can determine whether the node is working properly, which is a necessary precondition for keeping the high precision of all sensor data. Accordingly, the combination of temporal data-driven sleep scheduling and spatial data-driven anomaly detection can meet the core requirements on data of the tunnel monitoring system.

Spatial data-driven anomaly detection is implemented in the cluster head. The anomalies of sensor nodes mainly include “sensor error”, “abnormal data” and “disconnected node”. Although high precision sensors are useful for high precision data acquisition, sensors start to develop drift slowly as the network ages, which is a kind of systematic error. “Sensor error” including the systematic error and the random error is inevitable. “Abnormal data” can be caused by the unstable node or network. In addition “disconnected node” means a node is not a head or member of the cluster corresponding to the ring where this node is. These three anomalies have different features on data, like small deviation data in “sensor error”, big deviation data in “abnormal data” and no data in “disconnected node”.

To calculate the deviation data, which is the difference between the sensor data and the true data, Kriging is used to estimate the true data. Kriging, which is an excellent spatial interpolation method, can interpolate the sensor value at an unobserved location from observations of nearby locations. Compared with other spatial interpolation methods, Kriging has two main contributions for spatial data-driven anomaly detection in the tunnel monitoring system. (1) Kriging takes full account of the spatial correlation of sensor data to achieve high precision of spatial interpolation; (2) Kriging is applicable to the region where sampling data has characteristics of randomness and structural property. In addition, spatial data-driven anomaly detection in the tunnel monitoring system is exactly used in this kind of region. According to the inclination value *IV* = [*iv*_1_, *iv*_1_,…, *iv_m_*] of sensor nodes *X* = [*x*_1_, *x*_1_,…*x_m_*], Kriging can estimate the inclination angle iv^*^ at other locations through a linear combination of *iv*_1_, *iv*_1_,…, *iv_m_*, as shown in Equation (2):

(2) $$iv^{*}=\alpha^{*}+\sum_{k=1}^{m}\lambda_{k}\left(iv_{k}-\alpha_{k}\right),$$

where *α_k_* denotes the initial installation angle of sensor node *x_k_*, α* is the initial installation angle at other locations, *λ_k_* is the weight factor. Any inclination sensor node should be initialized after the installation, and the first sensor data of sensor node is used as its initial installation angle. The calculation of iv* is therefore based on the selection of *λ_k_*. These weight factors *λ_k_* are chosen by the unbiased condition given by Equation (3) and the minimum Kriging variance given by Equation (4).

(3) $$E\left[iv^{*}-iv\right]=\sum_{k=1}^{m}\lambda_{k}\left(iv_{k}-\alpha_{k}\right)-\left(iv-\alpha^{*}\right)=0,$$

(4) $$\text{min}\left[{\left(\delta^{*}\right)}^{2}\right]=Var\left(iv^{*}-iv\right)=\sum_{k=1}^{m}\sum_{j=1}^{m}\lambda_{k}\lambda_{j}c\left(iv_{k},iv_{j}\right)+Var\left(iv\right)-2\sum_{k=1}^{m}\lambda_{k}c\left(iv_{k},iv\right).$$

The *i*th cluster, which has seven sensor nodes *i*_1_, *i*_2_, *i*_3_, *i*_4_, *i*_5_, *i*_6_ and *i*_7_, is still selected as an example. The proposed anomaly detection is based on the cluster. All sensor nodes in a cluster are detected in turn. When the sensor node *i*_1_ is being detected, other nodes in this cluster are taken as the input of Kriging method, namely, *X* = [*x*_1_, *x*_1_, …, *x_m_*] = [*i*_2_, *i*_3_, *i*_4_, *i*_5_, *i*_6_, *i*_7_]. The true data of node *i*_1_ is estimated by Equations (2)–(4), and it is recorded as *iv*_1_^*^. Then, the deviation data of node *i*_1_ ∆*iv*_1_ is equal to |*iv*_1_ − *iv*_1_*|, where *iv*_1_ denotes the sensor data about inclination value of node *i*_1_.

Based on the deviation data, a simple anomaly classification and an anomaly indicator are presented. If the cluster head does not receive sensor data from a node, this node is in the state of “disconnected node”. If a node has the deviation data of which value is greater than the threshold value *β*, this node is in the state of “abnormal data”. Otherwise, this node is in the state of “sensor error”. The threshold value *β* can be set according to application scenarios of different sensor nodes. For inclination sensor node in the tunnel monitoring system, its threshold value *β* can be set to 1. Besides sensor node itself, some external factors can also cause “abnormal data”, like the artificial disturbance or major disasters. Whatever is causing “abnormal data”, they can increase the anomaly indicator *ξ* to arouse attention. Every sensor node has an anomaly indicator *ξ*, which is updated once ∆*iv*_1_ is updated, *ξ* = (*ξ* + ∆*iv*_1_)/2. The anomaly indicator *ξ* is divided into three levels including green, yellow and red. These levels of anomaly indicator are used to decide the priority level of node maintenance.

Data-driven anomaly detection based on the spatial correlation and Kriging method, as an important supplement to cooperative TDSS, makes the tunnel monitoring system own more practical value. Such anomaly detection also has two effects on the tunnel monitoring system: (1) the spatial correlation of sensor data is used to generate the anomaly indicator *ξ*, which can maintain and improve the precision of sensor data necessarily through node maintenance; (2) and data-driven anomaly detection can keep cooperative TDSS valid because keeping high precision of sensor data is a necessary precondition for achieving cooperative TDSS effectively.

**Figure 4:**
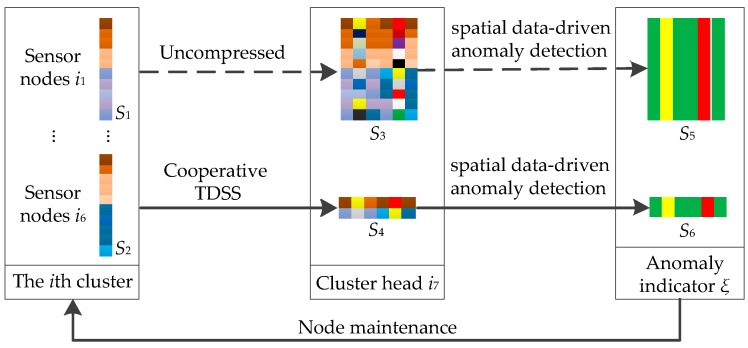
The data flow diagram of the *i*th cluster.

To better illustrate the relationship between cooperative TDSS and spatial data-driven anomaly detection, the data flow diagram of proposed methods is given. Since proposed methods are used in clustered WSNs, Figure 4 shows the data flow diagram of the *i*th cluster as an example. Colored squares are used to represent data. The rows of matrixes *S*_1_, *S*_2_, *S*_3_, *S*_4_, *S*_5_ and *S*_6_ represent space dimension (different sensor nodes), and the columns represent time dimension (different collecting time). The relationship between cooperative TDSS and spatial data-driven anomaly detection is divided into three parts: (1) cooperative TDSS ensures data synchronization for spatial data-driven anomaly detection. Cooperative TDSS realizes synchronous acquisition in the same ring, which is a necessary precondition for spatial data-driven anomaly detection; (2) cooperative TDSS reduces data redundancy to make spatial data-driven anomaly detection efficient. Compared with *S*_3_ that is uncompressed, *S*_4_ processed by cooperative TDSS preserves data with the key features to reduce data redundancy. In addition, anomalies are reflected in these data with the key features. Reducing unnecessary input data can therefore improve the efficiency of spatial data-driven anomaly detection; and (3) spatial data-driven anomaly detection can maintain and improve the precision of sensor data to keep cooperative TDSS valid. Accordingly, cooperative TDSS and spatial data-driven anomaly detection are interdependent with each other to improve common performance.

## 5. Performance Evaluation

This section presents the performance evaluation of temporal data-driven sleep scheduling and spatial data-driven anomaly detection through experiments and analysis.

### 5.1. Experiment Platform

**Figure 5:**
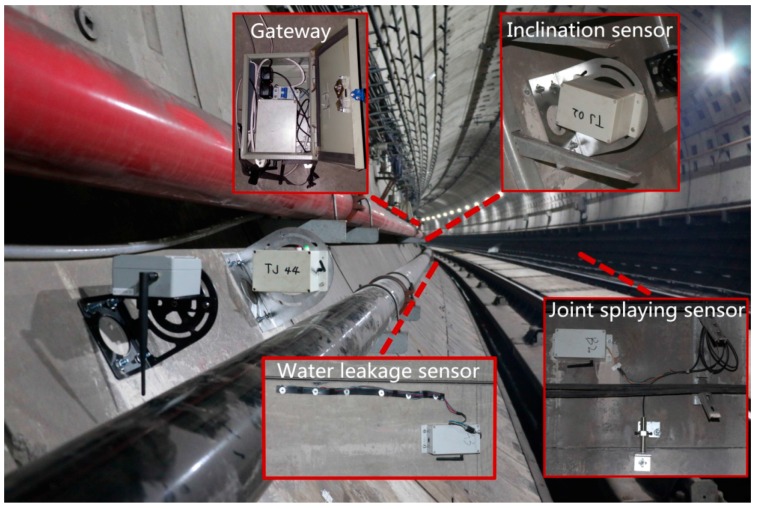
Tunnel monitoring system in Shanghai, China.

The proposed methods including temporal data-driven sleep scheduling and spatial data-driven anomaly detection are verified in an independently developed tunnel monitoring system in Shanghai, China, as shown in Figure 5. The sink node connects the sensor nodes through ZigBee (ZigBee Alliance, Davis, CA, USA), and it connects the server through 3G. This tunnel monitoring system, which is deployed from Tiantong Road Station to International Cruise Terminal Station on the Shanghai Metro #12 Line, has three kinds of sensor nodes including the inclination sensor, the water leakage sensor and the joint splaying sensor. Since the proposed methods use a kind of data-driven scheme, they can be applied to all kinds of sensor nodes. For the sake of clarity, experiments and analysis are based on the inclination sensor in this work.

The inclination angle of the segment in the tunnel changes slowly. The influence of the surrounding disturbance on the inclination sensor can be studied by analyzing inclination data in a short-term span. Inclination data changes very little in over a long-term span if no incident occurs. According to the experimental data, the variation scope of the axial inclination angle in a short-term span (several hours) with metro operation is less than 0.2° because metro train vibration causes instantaneous change in the inclination angle. In addition, the variation scope of the axial inclination angle over a long-term span (several months) without metro operation is less than 0.02°, because change in the inclination of the segment is extremely slow. Reducing the redundancy of data by the sleep scheduling is therefore significant. To verify the proposed methods in different time spans, experimental analysis in a short-term span and experimental analysis over a long-term span are given.

### 5.2. Experimental Analysis in a Short-Term Span

In metro tunnels, metro train vibration has a pronounced influence on inclination data, especially in a short-term span. Two experiment scenarios are therefore set in this section, including Scenario A: metro tunnel with metro operation (05:30–24:00) and Scenario B: metro tunnel without metro operation (0:00–5:30). In these two experiment scenarios, non-uniform sensing with cooperative TDSS under different *α* (0.01°, 0.05°) is performed, where the Default Acquisition Cycle (DRC) is set to 1 s. DRC is small so as to capture inclination data caused by metro train vibration.

**Figure 6:**
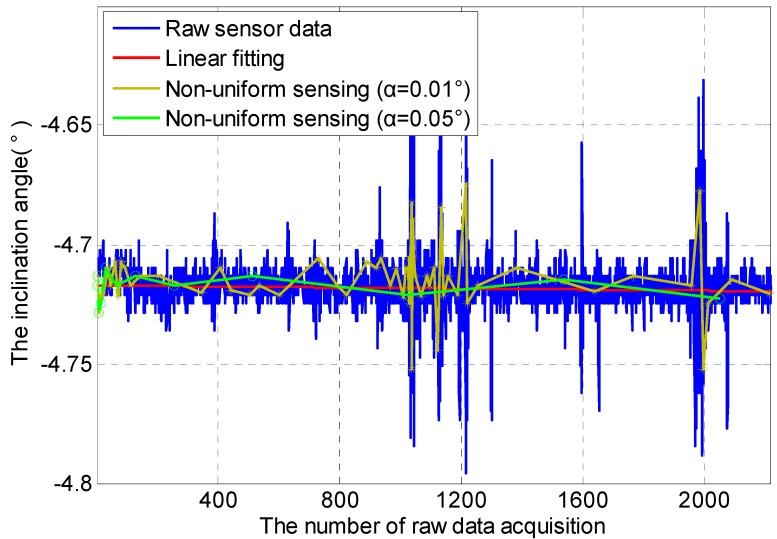
Non-uniform sensing with cooperative TDSS in Scenario A in a short-term span.

Non-uniform sensing with cooperative TDSS in Scenario A is shown in Figure 6. Inclination data has obviously instantaneous change at about 1100 DRC and 2000 DRC because metro train vibration causes instantaneous change in the inclination angle. The instantaneous change in inclination data at about 1600 DRC should be caused by the metro train nearby. Thus metro train vibration has a pronounced influence on inclination data in a short-term span. Monitoring the normal inclination angle of the segment in the tunnel is the real goal of such tunnel monitoring systems. Non-uniform sensing with cooperative TDSS can reduce sampling times by discarding unnecessary data. Under the condition that actual change in the inclination angle of the segment can be restored effectively through less data, the energy consumption of sensor nodes can be also reduced significantly by non-uniform sensing with cooperative TDSS. It can also be seen that the frequency of non-uniform sensing is inversely proportional to *α*. Non-uniform sensing with *α* = 0.01° shows that even instantaneous change in inclination data can be sensed if *α* is small enough. Sensing data using *α* = 0.01° are closer to the actual data than that using *α* = 0.05°, but larger energy consumption and more storage space are needed because of more sensing data. A suitable non-uniform sensing can therefore be realized by adjusting *α*.

**Figure 7:**
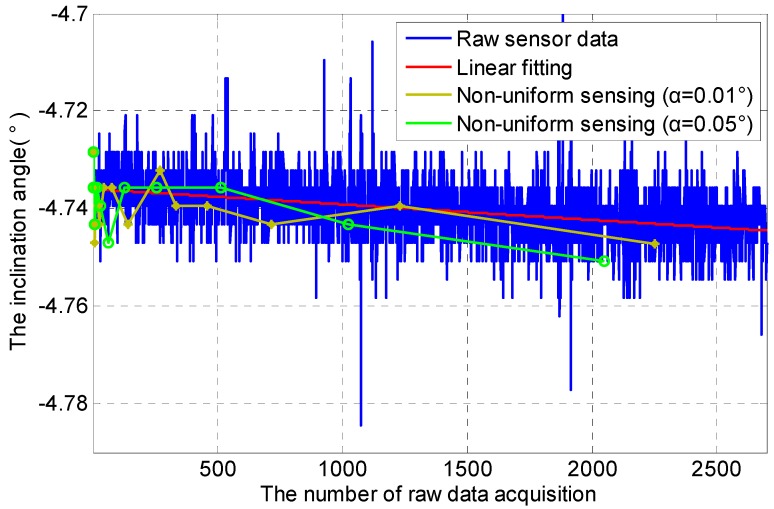
Non-uniform sensing with cooperative TDSS in Scenario B in a short-term span.

Figure 7 shows non-uniform sensing with cooperative TDSS in Scenario B. Without metro train vibration, inclination data is mainly affected by environmental noise. The variation scope of inclination data in Scenario B is smaller than that in Scenario A. Compared with Figure 6, the frequency of non-uniform sensing in Figure 7 is lower.

Consequently, cooperative TDSS can realize non-uniform sensing in a short-term span effectively. According to the temporal correlation, such non-uniform sensing based on cooperative TDSS can reduce the redundancy of data by discarding unnecessary data. In addition, actual change in the inclination angle of the segment can be restored effectively through less data. Moreover, the energy consumption of sensor nodes can be reduced significantly so that the life cycle of sensor nodes can be extended.

**Figure 8:**
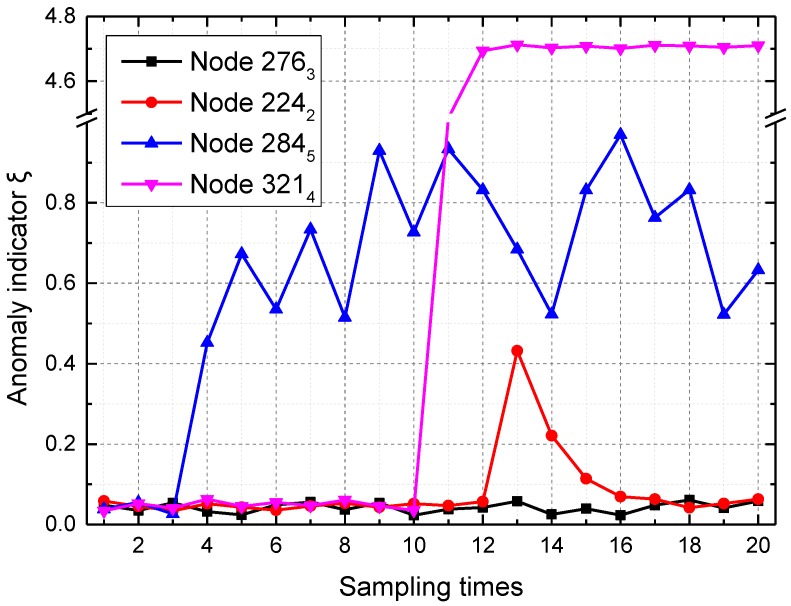
Anomaly indicator *ξ* of four sensor nodes in a short-term span.

Spatial data-driven anomaly detection is then verified. Figure 8 shows anomaly indicator *ξ* of four sensor nodes in a short-term span. Node 276_3_, which is a sensor node at the segment with the ring number 276, is working normally. Anomaly indicator *ξ* of node 224_2_ suddenly grows larger, and then decreases by about 50%, which means the anomaly “abnormal data” in node 224_2_ occurs once at the 13th sampling time. Anomaly indicator *ξ* of node 284_5_ suddenly grows larger, and then varies between 0.4 and 1 in an abnormal way, which means the anomaly “abnormal data” in node 284_5_ occurs continuously after the fourth sampling time. Anomaly indicator *ξ* of node 321_4_ suddenly grows larger, and then remains high at about 4.7, which is close to the value of inclination data. Thus, node 321_4_ is in the state of the anomaly “disconnected node” after the 11th sampling times. In a short-term span, “abnormal data” and “disconnected node” are the main anomalies.

In this tunnel monitoring system, anomaly indicator *ξ* is divided into three levels including green (0 ≤ *ξ* < 0.2), yellow (0.2 ≤ *ξ* < 0.5) and red (*ξ* ≥ 0.5).

**Figure 9:**
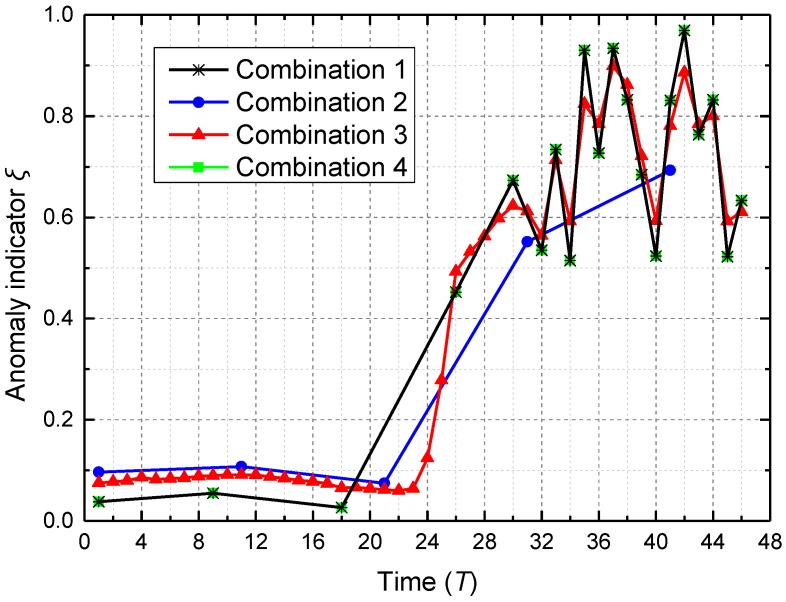
Anomaly indicator *ξ* of node 284_5_ using methods with different combinations in a short-term span.

To illustrate that the combination of temporal data-driven sleep scheduling and spatial data-driven anomaly detection can further improve system performance, four kinds of combinations are compared in node 284_5_ where the anomaly “abnormal data” occurs. Combination 1 denotes the combination of cooperative TDSS and spatial data-driven anomaly detection; Combination 2 denotes the combination of data acquisition with large cycle 10*T* (no TDSS) and spatial data-driven anomaly detection; Combination 3 denotes the combination of data acquisition with small cycle *T* (no TDSS) and spatial data-driven anomaly detection; Combination 4 denotes the combination of cooperative TDSS and spatial data-driven anomaly detection without node maintenance. As shown in Figure 9, compared with Combinations 2 and 3, Combination 1 has smaller anomaly indicator *ξ* when no anomaly occurs (time between *T* and 18*T*), and has a greater fluctuating range when an anomaly occurs (time between 26*T* and 46*T*). This means that cooperative TDSS can improve accuracy and sensitivity of spatial data-driven anomaly detection, which is realized by synchronous acquisition in the same ring. Sleep time of Combination 1 is inversely proportional to its anomaly indicator *ξ*, while Combinations 2 and 3 have constant sleep time, which is not related with anomaly indicator *ξ*. Compared with Combination 3, Combination 1 can detect the anomaly well by using longer sleep time when no anomaly occurs, which reduces the number of meaningless anomaly detections. Such meaningless anomaly detection not only increases the energy consumption, but also wastes limited computing capacity in WSN. Although Combination 2 has the largest sleep time, the effect of anomaly detection is bad when an anomaly occurs. Accordingly, cooperative TDSS can make spatial data-driven anomaly detection efficient by reducing data redundancy through non-uniform sensing. In a short-term span, Combinations 1 and 4 have the same ability in anomaly detection because enough time is required in node maintenance.

### 5.3. Experimental Analysis over a Long-Term Span

**Figure 10:**
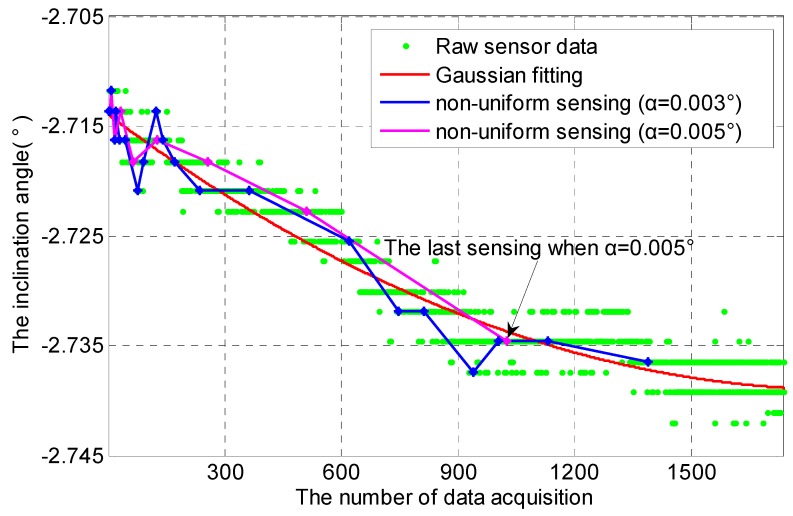
Non-uniform sensing with cooperative TDSS over a long-term span.

In the long-term monitoring of inclination of segments in the tunnel, DRC is set to one hour. Since sensor data is a kind of discrete data, Gaussian fitting is used to approximate the real changing trend of inclination angle. As shown in Figure 10, the curve of Gaussian fitting can approximate the real changing trend of inclination angle effectively, and its expression is given:

(5) $$Y=-2.739\times \text{exp}\left(-{\left(\frac{\left(x-1894\right)}{1.978\times 10^{4}}\right)}^{2}\right).$$

The variance *D_SC_* of sensor data to the curve of Gaussian fitting is defined as follows:

(6) $$D_{SC}=\frac{1}{n}\sum_{i=1}^{n}{\left(X_{i}-{\hat{X}}_{i}\right)}^{2},$$

where *X_i_* denotes the value of sensor data, and ${\hat{X}}_{i}$ denotes the value of the curve of Gaussian fitting. In Figure 10, *D_SC_* is equal to 0.00644, which is small. It is therefore reasonable that Gaussian fitting is used to approximate the real changing trend of inclination angle. By comparing the line of *α* = 0.003° and the line of *α* = 0.005°, non-uniform sensing with smaller *α* has a more number of data acquisition, and it is also more sensitive to the real inclination angle. However, it consumes more energy. If *α* is further reduced, the redundancy of sensor data will increased. The lines of *α* = 0.003° and *α* = 0.005° show that they can achieve a reasonable non-uniform sensing. *D_SC_*_003_ and *D_SC_*_005_ can be calculated by Equation (6), *D_SC_*_003_ = 4.3843 × 10^−6^, *D_SC_*_005_ = 2.9141 × 10^−6^, where *D_SCxxx_* (*x* is any natural number) denotes the variance of sensor data under *α* = 0.*xxx* to the curve of Gaussian fitting.

**Figure 11:**
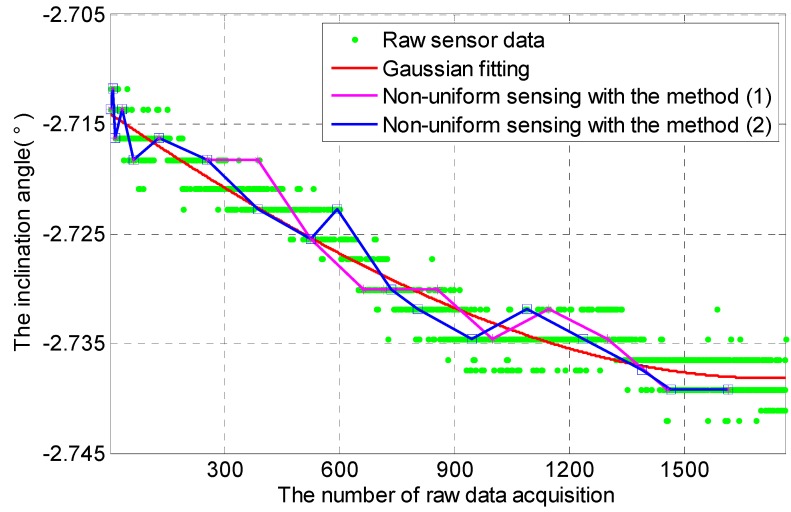
Non-uniform sensing with cooperative TDSS under two methods of calculating ∆*V*.

Two methods about calculating ∆*V*, which is the absolute value of difference between two sensor data, are made: (1) it is the absolute value of difference between two sensor data from the last two adjacent data acquisition; and (2) it is the absolute value of difference between the current sensor data and the last sensor data, of which ∆*V* is greater than or equal to *α*. As shown in Figure 11 where *α* = 0.003°, two methods can achieve a reasonable non-uniform sensing. *D_SC_* with method 1 and *D_SC_* with method 2 are equal to 3.7100 × 10^−6^ and 3.5245 × 10^−6^.

**Figure 12:**
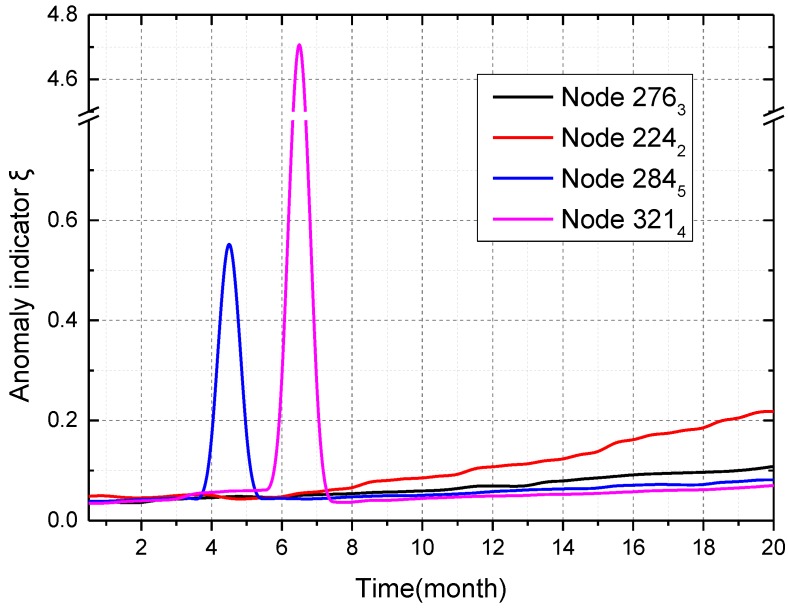
Anomaly indicator *ξ* of four sensor nodes over a long-term span.

Consequently, cooperative TDSS can also realize non-uniform sensing over a long-term span effectively. Spatial data-driven anomaly detection over a long-term span is then verified. As shown in Figure 12, node 284_5_, in which the anomaly “abnormal data” occurs according to the analysis in Figure 8, triggers the alarm level “red” after four months. Node 321_4_, in which the anomaly “disconnected node” occurs according to the analysis in Figure 8, triggers the alarm level “red” after six months. These two nodes are repaired by engineers in a timely way, and thus their anomaly indication *ξ* decrease after the reparation. As time passed by, sensor errors at different levels occur in four nodes, which are mainly caused by the sensor drift. It can be seen that the anomaly “sensor error” mainly occurs in nodes over a long-term span. After eight months, their anomaly indication *ξ* increase slowly, and *ξ* of node 224_2_ is larger than that of the other three nodes. Node 224_2_ triggers the alarm level “yellow” after 19 months, which will be considered in the node maintenance. Since anomaly indication *ξ* is inversely proportional to the precision of sensor data, spatial data-driven anomaly detection can maintain and improve the precision of sensor data effectively.

**Figure 13:**
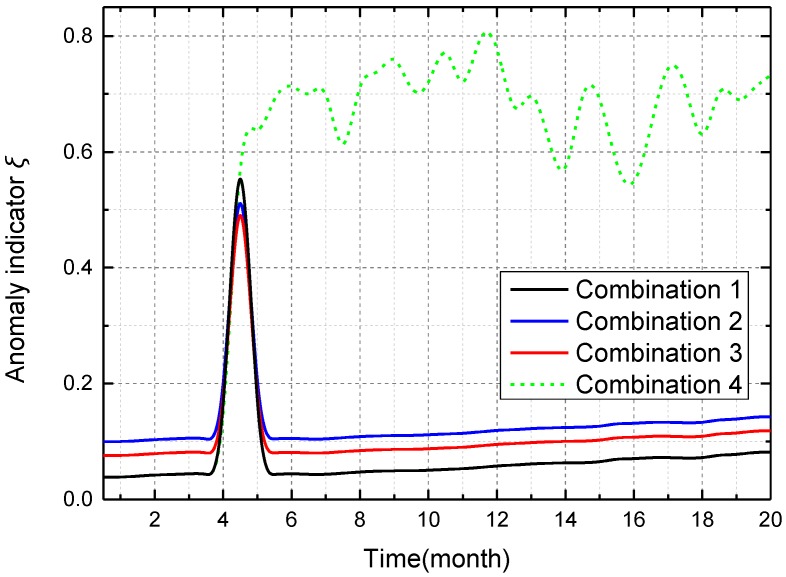
Anomaly indicator *ξ* of node 284_5_ using methods with different combinations over a long-term span.

Four kinds of combinations are also compared in node 284_5_ over a long-term span. As shown in Figure 13, anomaly indicator *ξ* of Combination 1 is smaller than that of Combinations 2 and 3 when anomaly indicator *ξ* has the level of green. This means that cooperative TDSS can improve accuracy of spatial data-driven anomaly detection, which is realized by synchronous acquisition in the same ring. Because the node is repaired by engineers in a timely way, anomaly indication *ξ* of Combinations 1–3 decrease after the reparation. Once a failure occurred in the node, Combination 4 would not repair it through node maintenance; thus, anomaly indicator *ξ* of Combination 4 would always change irregularly. Such change in anomaly indicator *ξ* means that sensor data always obviously change, although no event occurs in the tunnel. Such change in sensor data inevitably leads to little sleep time, which increases the unnecessary energy consumption. Since TDSS is a kind of data-driven mechanism, the high precision of sensor data should be ensured. When failure exists in the node for a long time, cooperative TDSS cannot achieve appropriate sleep time for the real condition in the tunnel. Accordingly, spatial data-driven anomaly detection can maintain and improve the precision of sensor data through node maintenance to keep cooperative TDSS valid.

## 6. Conclusions

In this work, we exploit the spatial–temporal correlation of sensor data to improve the performance of the tunnel monitoring system based on clustered WSN. The combination of temporal data-driven sleep scheduling and spatial data-driven anomaly detection is proposed. The proposed temporal data-driven sleep scheduling model is inspired by TCP congestion control. By exploiting the temporal correlation of sensor data, TDSS achieves appropriate sleep time to reduce the temporal redundancy of sensor data. Since the sleep time increases exponentially in the initial phase, such appropriate sleep time can be achieved quickly.

Based on long and linear cluster structure built for the tunnel monitoring system, cooperative TDSS and spatial data-driven anomaly detection are proposed. Cooperative TDSS in the cluster not only realizes an efficient data-driven sleep scheduling based on the temporal correlation to reduce the energy consumption, but can also realize synchronous acquisition in the same ring for analyzing the situation of the every ring. Different from most existing works where the spatial correlation is used to further compress data, this work uses the spatial correlation to achieve cluster-based anomaly detection. The anomalies of sensor nodes mainly include “sensor error”, “abnormal data” and “disconnected node”. Spatial data-driven anomaly detection in the cluster is realized by using Kriging method based on the spatial correlation.

The performance of temporal data-driven sleep scheduling and spatial data-driven anomaly detection is evaluated in experiments on the Shanghai Metro #12 Line. The experiments’ results show that cooperative TDSS can realize non-uniform sensing in a short or long-term span effectively. Such non-uniform sensing based on cooperative TDSS not only can reduce the energy consumption of sensor nodes significantly but also can restore actual change in the inclination angle of the segment effectively through less data. It is also verified in the experiments that spatial data-driven anomaly detection, which generates an anomaly indicator *ξ* to decide the priority level of node maintenance, can maintain and improve the precision of sensor data effectively, especially over a long-term span.

## References

[1] Aygün B., Gungor V.C. (2011). Wireless sensor networks for structure health monitoring: Recent advances and future research directions. Sens. Rev..

[2] Sayyed A., de Araújo G.M., Bodanese J.P., Becker L.B. (2015). Dual-stack single-radio communication architecture for UAV acting as a mobile node to collect data in WSNs. Sensors.

[3] He B., Li Y., Huang H., Tang H. (2014). Spatial–temporal compression and recovery in a wireless sensor network in an underground tunnel environment. Knowl. Inf. Syst..

[4] Chen Z., Ranieri J., Zhang R., Vetterli M. (2015). DASS: Distributed adaptive sparse sensing. IEEE Trans. Wirel. Commun..

[5] Al-Kahtani M.S. (2015). Efficient Cluster-Based Sleep Scheduling for M2M Communication Network. Arab. J. Sci. Eng..

[6] He B., Li G. (2014). PUAR: Performance and usage aware routing algorithm for long and linear wireless sensor networks. Int. J. Distrib. Sens. Netw..

[7] Tyagi S., Kumar N. (2013). A systematic review on clustering and routing techniques based upon LEACH protocol for wireless sensor networks. J. Netw. Comput. Appl..

[8] Dhasian H.R., Balasubramanian P. (2013). Survey of data aggregation techniques using soft computing in wireless sensor networks. IET Inf. Secur..

[9] Alsheikh M.A., Lin S., Niyato D., Tan H.-P. (2016). Rate-distortion Balanced Data Compression for Wireless Sensor Networks. IEEE Sens. J..

[10] Arunraja M., Malathi V., Sakthivel E. (2015). Energy conservation in WSN through multilevel data reduction scheme. Microprocess. Microsyst..

[11] Gong B., Cheng P., Chen Z., Liu N., Gui L., de Hoog F. (2015). Spatiotemporal compressive network coding for energy-efficient distributed data storage in wireless sensor networks. IEEE Commun. Lett..

[12] Ma S., Qian J., Sun Y. (2013). Optimal sleep scheduling scheme for wireless sensor networks based on balanced energy consumption. J. Comput..

[13] Yang O., Heinzelman W. (2013). An adaptive sensor sleeping solution based on sleeping multipath routing and duty-cycled MAC protocols. ACM Trans. Sens. Netw. TOSN.

[14] Liu F., Tsui C.Y., Zhang Y.J. (2010). Joint routing and sleep scheduling for lifetime maximization of wireless sensor networks. IEEE Trans. Wirel. Commun..

[15] Titouna C., Aliouat M., Gueroui M. (2015). Outlier detection approach using bayes classifiers in wireless sensor networks. Wirel. Pers. Commun..

[16] Nisha U.B., Maheswari N.U., Venkatesh R., Abdullah R.Y. (2015). Improving Data Accuracy Using Proactive Correlated Fuzzy System in Wireless Sensor Networks. KSII Trans. Internet Inf. Syst..

[17] Xiang Y., Xuan Z., Tang M., Zhang J., Sun M. (2016). 3D space detection and coverage of wireless sensor network based on spatial correlation. J. Netw. Comput. Appl..

[18] Elson J., Girod L., Estrin D. (2002). Fine-grained network time synchronization using reference broadcasts. ACM SIGOPS Oper. Syst. Rev..

